# Rapid recurrence of stage IIB non-gestational ovarian choriocarcinoma with minor yolk sac tumor: A rare case report and literature review

**DOI:** 10.1016/j.gore.2023.101312

**Published:** 2023-11-30

**Authors:** Edward K. Maybury, Nnamdi I. Gwacham, Charanjeet Singh, Susan Mondo, Sarfraz Ahmad, Nathalie D. McKenzie

**Affiliations:** a Kansas City University, Kansas City, MO 64106, USA; b AdventHealth Cancer Institute, Gynecologic Oncology Program, Orlando, FL 32804, USA; c AdventHealth Orlando, Department of Pathology, Orlando, FL 32804, USA

**Keywords:** Non-gestational ovarian choriocarcinoma, Fertility-sparing laparoscopic debulking surgery, Yolk-sac tumor, Case report, Literature review

## Abstract

Non-gestational ovarian choriocarcinoma (NGOC) is a rare phenomenon seldom reported in the literature. Patients often present with abdominopelvic pain, and sometimes a palpable adnexal mass. Surgical excision is paramount in treating this malignancy; however, fertility-preserving care is a debated topic among gynecologic oncologists. Most patients reported in the literature are nulliparous women of child-bearing age. It is important to consider fertility preservation whilst balancing oncologic outcomes. We present a case of an 18-year-old nulliparous female with stage IIB NGOC that had disease progression in the lungs and pelvis shortly after undergoing fertility-preserving surgery. She required emergent completion surgery and received etoposide (E), methotrexate (M), actinomycin-D (A), cyclophosphamide (C) and vincristine (O) (EMA-CO) with complete response. She remains disease-free after 21-months.

## Highlights

• Non-gestational ovarian choriocarcinoma (NGOC) is rare.
• NGOC can behave aggressively with poor survival and high recurrence rates.
• Expert pathology evaluation is warranted given its rarity.
• Individualized care and close monitoring is crucial for NGOC management.
• Fertility preservation for NGOC is feasible in select cases.

## 1 Introduction

With an incidence of less than 0.6 % of malignant ovarian tumors, non-gestational ovarian choriocarcinoma (NGOC) is both aggressive and extremely rare (Cronin et al., 2022). It necessitates special attention due to its close resemblance in presentation to gestational ovarian choriocarcinoma (GOC). They often present in a similar manner to ectopic pregnancies, with pelvic pain, abnormal bleeding, and elevated β-human chorionic gonadotropin (β-hCG). Tissue analysis for lack of paternal genetic components effectively makes the diagnosis of NGOC. Initial evaluation typically involves: a comprehensive gynecologic history, evaluating fertility potential, obtaining β-hCG levels, and ultrasound imaging. Once other differential diagnoses are ruled out, and NGOC is suspected, comprehensive computed tomography (CT) scans will assist in ruling out metastasis in the liver, lungs, and pelvis. Treatment often requires surgical resection and chemotherapy; most commonly utilizing Bleomycin, Etoposide, and Cisplatin (BEP) (Cronin et al., 2022). With a timely response to initial presentation, patients can be offered fertility sparing options with potential long-term survival (Cronin et al., 2022). Close monitoring and long-term surveillance following surgery and chemotherapy are important conclusive steps. Most patients are fortunate to have positive outcomes with an ability to maintain fertility.

## 2 Case

**Fig. 1:**
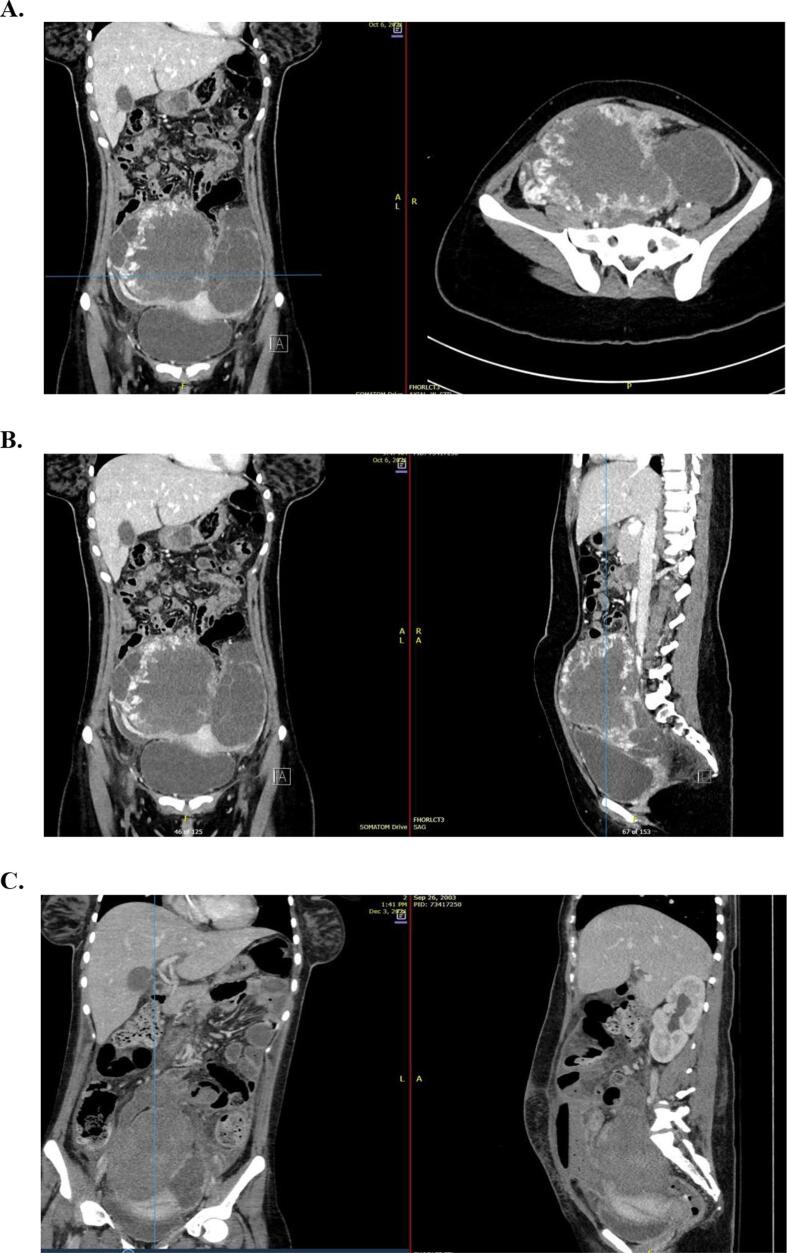
**(A)** Computed tomography (CT) anterior/posterior (A/P) coronal and transverse. Multiple complex cystic masses in both adnexa measuring up to 15.5 cm and 13.6 cm. **(B)** CT A/P coronal and sagittal. Multiple complex cystic masses in both adnexa. **(C)** CT A/P coronal and sagittal. Peritonitis secondary to bowel rupture from tumor invasion to the sigmoid colon.

**Fig. 2:**
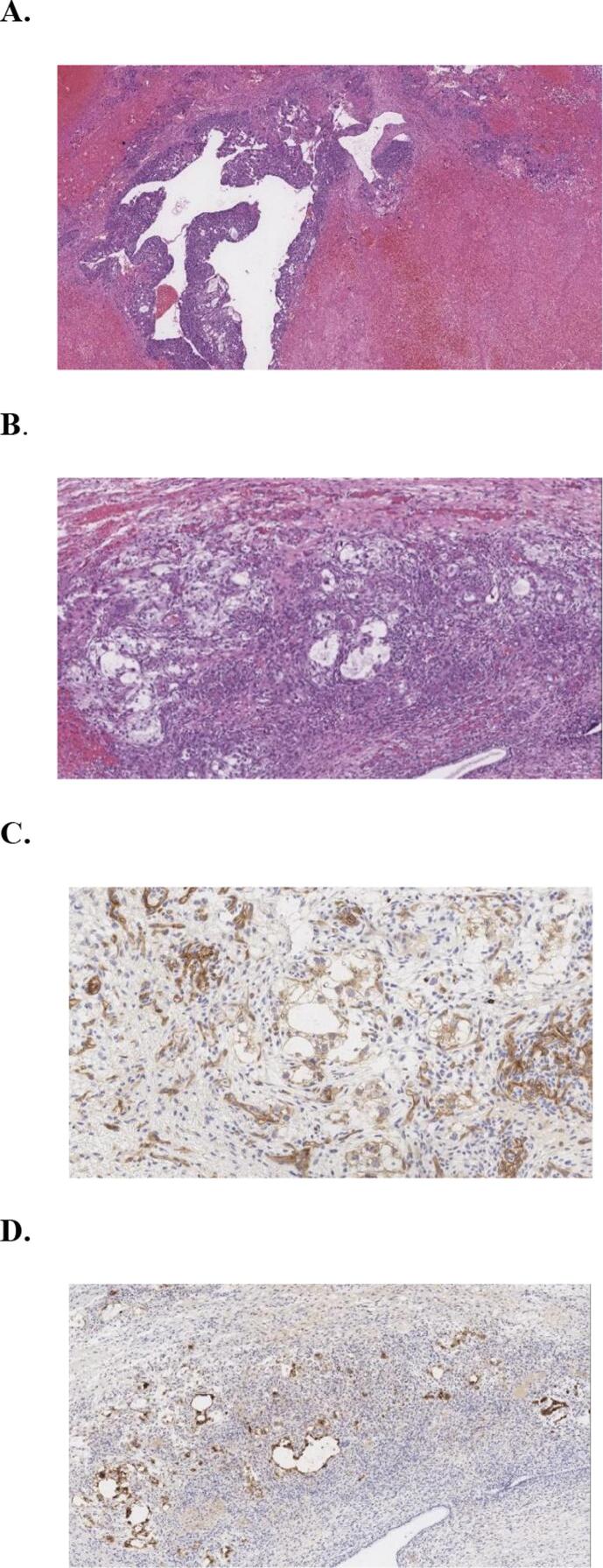
**(A)** Choriocarcinoma hematoxylin and eosin (H&E) Original magnified 20x. **(B)** Minor yolk sac component H&E original magnified 40x. **(C)** Minor yolk sac component, cluster differentiation (CD)117 stain, original magnified 100x. **(D)** Minor yolk sac component, Gylpican-3 stain, original man Gnified 40x.

An 18-year-old nulliparous female presented to the emergency department (ED) with constipation and abdominal pain for the past month. Upon presentation, β-hCG was > 100,000 mIU/mL. She denied ever being sexually active and reported a history of irregular menstrual cycles. Pelvic ultrasound demonstrated a 13 cm pelvic mass, with theca-lutein cysts, and without an associated intrauterine pregnancy. Further investigation with CT of the chest, abdomen, and pelvis revealed multiple complex cystic masses in both adnexa measuring up to 15.5 cm and 13.6 cm as well as several small nodular peritoneal implants [Fig. 1 **A, B, C**], [Fig. 2 **A, B, C, D**].

Fertility-preservation with exploratory laparotomy, unilateral salpingo-oophorectomy, peritoneal biopsies, total omentectomy, and pelvic and aortic lymphadenectomy was completed, revealing a stage IIB non-gestational ovarian choriocarcinoma with a minor yolk-sac tumor component and dysgerminoma.

During her recovery, barely one-month after surgery, she presented to the ED with an acute abdomen and findings of peritonitis secondary to bowel rupture from recurrent tumor invasion to the sigmoid colon **[**Fig. 1**C]**. At that time, she underwent an urgent laparotomy inclusive of a complete modified radical hysterectomy, abdominal washout, with en-bloc unilateral salpingo-oophorectomy, rectosigmoid resection and descending colostomy. She experienced a 9-day intensive care unit (ICU) stay during which she required vasopressors, parenteral nutrition, antimicrobials, and blood transfusions. She was ultimately discharged home on post-operative day-18 after she met all her milestones for discharge. Prior to initiation of chemotherapy, her β-hCG was down to 83 mIU/mL. She ultimately went on to complete 8-cycles of EMA-CO without radiographic findings of disease recurrence. She had repeat β-hCG levels every month that remained undetectable during the surveillance period. She eventually had her colostomy reversed and remains disease free after 21-months.

## 3 Methods

An extensive literature search for peer-reviewed articles written in the English language was performed, keywords such as non-gestational ovarian choriocarcinoma and fertility-preserving surgery were primarily used to review/search for publications through Google, PubMed, and the NIH National Library of Medicine. Further article retrieval was obtained from the references of well-cited publications, and a relevant list of cases was formed **[**Table 1**]** [see also Supplement 1 **-** for Extra List of References].

**Table 1:** Treatment and outcomes of known cases of NGOC from the year 1995 to present. [see also Supplement 1 **-** for Extra List of References].

**Author(s)**	**Publi-cation Year**	**Age (Year)**	**β-hCG** **[mIU/mL]**	**Diagnosis**	**Surgical Tx**	**Chemotherapy**	**F/U Time / Outcomes**
**Byeun** (Byeun et al., 1995)	**1995**	**28**	**13,378**	**NGOC**	**TAH w/ BSO and partial LND**	**BEP**	**Unk / DF**
Trigueros Velazquez*	1995	21	200,000	NGOC	TAH w/ BSO, R hemicolectomy	Cisplatin, bleomycin, and vinblastine	48 mos / DF
**Inaba** (Inaba et al., 2000)	**2000**	**12**	**25,000**	**Stage III NGOC w/ dissemination and lung mets**	**RSO, LOC, omentectomy**	**BEP, autologous bone marrow stem cell transplantation**	**Unk / DF**
Jain*	2000	33	146	Mixed NGOC	RSO	–	18 mos / DF
**Shigematsu** (Shigematsu et al., 2000)	**2000**	**45**	**–**	**NGOC**	**–**	**Chemotherapy (unk)**	**Unk / died**
**Goswami** (Goswami et al., 2001)	**2001**	**18**	**88,385**	**NGOC w/ contralateral teratoma**	**LSO, omental and peritoneal biopsies**	**Mitoxantrone and chlorambucil**	**5 mos / DF**
Ozaki*	2001	54	–	Mixed NGOC	Unk surgery	–	Unk / died
Vimala*	2002	24	10,000	Mixed NGOC	RSO	–	18 mos / DF
**Tsujioka** (Tsujioka et al., 2003)	**2003**	**19**	**–**	**NGOC**	**–**	**EMA-CO**	**Unk / Unk**
**Balat** (Balat et al., 2004)	**2004**	**24**	**8,968**	**NGOC**	**TAH w/ BSO, partial omentectomy, sternum mass excision**	**BEP**	**Unk / died**
Bazot*	2004	38	2,460,000	NGOC	TAH w/ BSO	Chemotherapy (unk)	84 mos / DF
**Ozdemir** (Ozdemir et al., 2004)	**2004**	**13**	**91,028**	**NGOC**	**RSO**	**MAC**	**9 mos / DF**
**Coracki** (Corakçi et al., 2005)	**2005**	**22**	**15,050**	**NGOC**	**TAH w/ BSO and partial LND**	**BEP**	**12 mos / DF**
**Koo** (Koo et al., 2006)	**2006**	**33**	**–**	**NGOC**	**Unk surgery**	**Chemotherapy (Unk)**	**17 mos / DF**
**Gerson** (Gerson et al., 2007)	**2007**	**33**	**564,000**	**NGOC w/ spleen mets**	**RSO, then TAH w/ LSO, then splenectomy**	**EMA-CO**	**12 mos / DF**
**Roghaei** (Roghaei et al., 2007)	**2007**	**47**	**970**	**NGOC**	**TAH w/ BSO, partial omentectomy, pelvic LND**	**EMA-CO**	**Unk / DF**
**Yamamoto** (Yamamoto et al., 2007)	**2007**	**19**	**206,949**	**NGOC**	**Left oophorectomy**	**EMA**	**12 mos / DF**
**Kong** (Kong et al., 2009)	**2009**	**10**	**–**	**NGOC**	**Unk surgery**	**Cisplatin**	**Unk / DF**
Park*	2009	55	64,838	NGOC w/ lung mets	TAH w/ BSO	BEP	20 mos / DF
Butler*	2010	24	15,000	NGOC	RSO	–	Unk / unk
Gon*	2010	21	279,000	NGOC	RSO	–	Unk / unk
Gremeau*	2010	46	4,962	Stage IV NGOC	LSO, peritoneal biopsy	–	Unk / died
Gremeau*	2010	26	1,183	Mixed NGOC and GOC	LSO, peritoneal biopsy	–	12 mos / DF
Hafezi- Bakhtiari*	2010	65	332,966	Mixed NGOC	BSO, LND, omentectomy, partial anterior peritonectomy, partial bladder resection	BEP	Unk / died
Hu*	2011	23	26,516	NGOC	Tumor resection (unk)	BEP	30 mos / unk
Lee*	2011	12	5,823	NGOC	LSO	–	25 mos / DF
Lv*	2011	48	7,664	NGOC	TAH w/ BSO, omentectomy, appendectomy, aortic LN biopsies, peritoneal biopsies	BEP	12 mos / DF
Aggarwal*	2012	15	–	Stage III NGOC	LSO, omentectomy, appendectomy	–	Unk / unk
Nikolic*	2012	20	–	Mixed stage IV NGOC and GOC	LSO, lumpectomy	Methotrexate + cyclophosphamide prior to Sx.	1 mos / died
Choi*	2013	33	74,612	NGOC	LSO, ROC, peritoneal biopsies, endometrial biopsies	EMA	60 mos / DF
Exman*	2013	24	675,713	NGOC	TAH w/ BSO, omentectomy	BEP prior to Sx	1 mos / DF
Goyal*	2014	18	3,751	Mixed NGOC and GOC	RSO	BEP	6 mos / DF
Heo*	2014	12	20,257	Stage IA NGOC	LSO	BEP	14 mos / DF
Wan*	2014	54	291,116	NGC of the salpinx	TAH w/ BSO	5-Fluorouracil and kengsengmycin (KSM)	14 mos / DF
Haruma*	2015	19	373,170	NGOC w/ lung mets	LSO	EMA-CO	10 mos / DF
Hayashi*	2015	10	6,600	NGOC	RSO	BEP	62 mos / DF
Rao*	2015	26	8,160	NGOC w/ brain mets	RSO, partial omentectomy, partial splenectomy, R adrenalectomy	6-cycles of bleomycin, etoposide, and vincristine	14 mos / DF
Xin*	2015	23	18,000	Stage IIB NGOC	LOC, LSO, omentectomy, peritoneal biopsy, retroperitoneal LND	BEP	9 mos / DF
Ahn*	2016	41	–	NGOC	Hand-assisted laparoscopic staging surgery	Chemotherapy (Unk)	48 mos / DF
Koyanagi*	2016	29	10,800	Mixed NGOC w/ lung mets	TAH w/ RSO, peritoneal resection nodule biopsy, lung biopsy	–	Unk / Unk
Wang*	2016	13	2,045 then 79,102	NGOC w/ disseminated mets	TAH w/ LSO, partial R oophorectomy	Cisplatin, bleomycin, and vincristine	4 mos / Died
Wang*	2016	13	–	NGOC	LSO	–	12 mos / Unk
Xing*	2016	13	2,334	NGOC	–	–	Unk / Unk
Ghalleb*	2017	23	–	Stage IIIC Yolk Sac Tumor w/ peritoneal mets	–	BEP	62 mos / DF
Jia*	2017	27	200,000	GOC	ROC	EP-EMA	32 mos / DF
Mascilini*	2017	27	22,000	NGOC	LSO	BEP	60 mos / DF
Syed*	2017	38	300,000	NGOC	TAH w/ BSO, omentectomy	BEP	3 mos / DF
Ahmad*	2018	16	–	NGOC	Unilateral oophorectomy	Chemotherapy (Unk)	12 mos / DF
Ahmad*	2018	16	–	NGOC w/ mets	Partial oophorectomy	Chemotherapy (Unk)	Unk / Unk
Kumar*	2018	34	877,414	NGOC w/ jejunal mets	Resection of small bowel only	–	6 mos / DF
Yang*	2019	14	764,826	NGOC	RSO. 1 mos later, TAH w/ omentectomy	EMA-CO. 1 mos later, vincristine, actinomycin-D, etoposide, and fluorouracil	12 mos / DF
Peng*	2020	12	120,420	NGOC w/ lung mets	RSO	Actinomycin and etoposide, then EMA-CO prior to Sx	3 mos / DF
Sadiq*	2020	53	–	NGOC	TAH w/ BSO, peritoneal tumor debulking	Paclitaxel + carboplatin	2 mos / Died
**Adow** (Adow et al., 2021)	**2021**	**25**	**1,000,000**	**NGOC**	**TAH w/ BSO**	**BEP**	**12 mos / DF**
Lee*	2021	15	–	Stage IIA NGOC	Right oophorectomy, omentectomy, and peritoneal washing cytology	–	15 yrs / DF
Nishino*	2021	30 s	5,030	NGOC w/ lung mets	TAH w/ BSO, L lung segmentectomy	EMA, paclitaxel, and cisplatin. Then, fluorouracil and actinomycin-D. Then, EMA-CO. Then, BEP	21 mos / Died
Yee*	2021	16	624,177	Stage IV NGOC w/ lung and liver mets	LOC, partial left oophorectomy	–	1 mos / Died
Our case	–	18	100,000	Stage IIB NGOC w/ yolk sac tumor, lung mets, and colon mets	USO, omentectomy, pelvic + aortic LN biopsies, peritoneal biopsies. Then, TAH w/ USO, proctosigmoidectomy	EMA-CO	15 mos / DF
*Abbreviations:* β-hCG - Beta-human chorionic gonadotropin; F/U = Follow-up; NGOC = Non-gestational ovarian choriocarcinoma; LSO/RSO = Left/Right salpingo-oophorectomy; BSO = Bilateral salpingo-oophorectomy; LN = Lymph node; LND = Lymph node dissection; TAH = Total abdominal hysterectomy; LOC/ROC = Left/Right ovarian cystectomy; BEP = bleomycin, etoposide, + cisplatin; EMA = etoposide, methotrexate, + actinomycin-D; EMA-CO = etoposide, methotrexate, actinomycin-D, cyclophosphamide, + vincristine; MAC = methotrexate, actinomycin-D, + cyclophosphamide; EP-EMA = etoposide + cisplatin, then EMA; DF = Disease-free; Unk = unknown; mos = Months; Mets = Metastasis; w/ = With.							
* References in un-bold font are available in Supplement 1 file (due to limited references allowed in the main document).							

Cases have been excluded because of the lack of relevance to our reported case. Reasons include tumors located in the female reproductive system that were not NGOC or GOC in nature. Cases dated prior to the year 1995 were also excluded to highlight relatively modern efficacious chemotherapy methods, and the trends in accepted treatments that more closely resemble currently available options.

## 4 Discussion

NGOC is a highly aggressive neoplasm, but it is also rare. Only 57 cases have been reported in the peer-reviewed literature since the year 1995, with ever-increasing achievements in maintaining fertility. Chemotherapy with BEP is the most utilized standard of care after fertility preserving surgery and is associated with a high response rate (Cronin et al., 2022). Fertility-sparing surgery for early-stage disease is a reasonable first option, given that the peak incidence of NGOC occurs during early reproductive years (Adow et al., 2021). More radical surgical approaches are required in advanced stages in combination with well-studied adjunct chemotherapy regimens.

### 4.1 Outcomes

Of the 58 known cases (including our own), 38 (66.6 %) cases were known to be disease-free following surgery and/or chemotherapy, 10 (17.5 %) cases died, and nine (15.8 %) cases were lost to follow-up **[**Table1**]** [see also Supplement 1 **-** for Extra List of References]. *Surveillance:* The β-hCG levels are a relatively poor prognostic indicator. Our literature review provided a limited number of cases that reported these numbers. The average β-hCG levels among deceased patients was 175,868 mIU/mL (n = 6) and among the disease-free patients, it was 265,952 mIU/mL (n = 32). An extremely limiting factor regarding β-hCG is the timing at which the levels were acquired. Some cases tracked levels after chemotherapy had begun, and others did not track them at all. Follow-up levels of zero are more helpful with a prior elevated β-hCG level to compare with.

### 4.2 Treatment

An emphasis on fertility-sparing treatment options was imperative for this patient population. 53 out of the 58 cases (91.3 %) were younger than 51; the average age of menopause. It is unclear how many of these patients endorsed a desire to maintain fertility, or how many children each respective patient already had. In any case, this is a portion of the risks that ought to be discussed early on in a setting of shared decision-making. Of the 38 surgical cases, 16 (42.1 %) necessitated total abdominal hysterectomy with bilateral salpingo-oophorectomy. The remaining 22 cases (57.9 %) were able to maintain some form of fertility. Of the 37 cases in which the specific chemotherapy treatment was reported, including our case, 17 (45.9 %) used BEP alone, five (13.5 %) used EMA-CO alone, and 15 (40.5 %) used another combination modality.

### 4.3 Prognostic factors

Of the 39 cases known to be disease free, 32 (82.1 %) utilized both surgery and chemotherapy. Of the 10 cases known to have died, six (60 %) also utilized both methods. There is not enough evidence to draw conclusions based on these numbers, but the dual approach to treating NGOC is the most commonly demonstrated. Fourteen (82.4 %) patients treated with BEP alone survived with two deaths and one unknown outcome. Four patients treated with EMA-CO alone survived (80 %), with one unknown outcome. Eleven patients treated with another combination modality survived (73.3 %), with four deaths. More details can be found in Table 1 [see also Supplement 1 **-** for Extra List of References].

## 5 Conclusion

NGOC is an exceptionally rare and aggressive ovarian malignancy, necessitating a careful balance between appropriate treatment and maintenance of fertility. It is difficult to distinguish, and a delay in diagnosis can increase the possibility of necessitating more aggressive surgical resection. The majority of patients affected by NGOC will likely be women of child-bearing age; therefore, their outcomes in prognosis and fertility are best when assessed early on and a shared treatment plan can begin as soon as possible.

## Precis

Primary and recurrent non-gestational ovarian choriocarcinomas are rare with generally poor prognosis; however, opportunities exist to optimize cancer control.

## Consent

Informed consent was obtained from the patient for publication of this case report and accompanying images.

## Funding

This research case report was not supported by any external funds.

## Declaration of competing interest

The authors declare that they have no known competing financial interests or personal relationships that could have appeared to influence the work reported in this paper.
