# Supplementary material for: Rapid recurrence of stage IIB non-gestational ovarian choriocarcinoma with minor yolk sac tumor: A rare case report and literature review
Source: Gynecol Oncol Rep. 2023 Nov 30;50:101312. doi: 10.1016/j.gore.2023.101312 (PMC10703604; doi:10.1016/j.gore.2023.101312)
Supplement: Supplementary data 1 [file mmc1.docx]

**Supplement 1 – Extra List of References (Maybury EK, *et al*)**

1. Aggarwal A, Ocon AJ, Nibhanipudi K. A 15-year-old female with amenorrhea, abdominal distention, and elevated human chorionic gonadotropin: pregnancy, right? Not so fast. Pediatr Emerg Care. 2012;28(10):1057-9. doi: 10.1097/PEC.0b013e31826ce9d0.
2. Ahmad MF, Abu MA, Chew KT, Sheng KL, Zakaria MA. A positive urine pregnancy test (UPT) with adnexal mass; ectopic pregnancy is not the ultimate diagnosis. Horm Mol Biol Clin Investig. 2018;34(2):/j/hmbci.2018.34.issue-2/hmbci-2018-0004/hmbci-2018-0004.xml.
3. Ahn SH, Roh HJ, Cho HJ, You SG, Lee SH, Kwon YS. Pure non-gestational choriocarcinoma arising in the ovary. Eur J Gynaecol Oncol. 2016;37(4):549-553.
4. Bazot M, Cortez A, Sananes S, Buy JN. Imaging of pure primary ovarian choriocarcinoma. AJR Am J Roentgenol. 2004;182(6):1603-4. doi: 10.2214/ajr.182.6.1821603.
5. Butler R, Chadha Y, Davies J, Singh M. A case of primary tubal gestational choriocarcinoma. Aust N Z J Obstet Gynaecol. 2010;50(2):200-1. doi: 10.1111/j.1479-828X.2009.01124.x.
6. Choi YJ, Chun KY, Kim YW, Ro DY. Pure non-gestational choriocarcinoma of the ovary: A case report. World J Surg Oncol. 2013;11:7. doi: 10.1186/1477-7819-11-7.
7. Exman P, Takahashi TK, Gattás GF, Cantagalli VD, Anton C, Nalesso F, Diz Mdel P. Primary ovary choriocarcinoma: individual DNA polymorphic analysis as a strategy to confirm diagnosis and treatment. Rare Tumors. 2013;5(2):89-92. doi: 10.4081/rt.2013.e24.
8. Ghalleb M, Bouzaiene H, Slim S, Hadiji A, Hechiche M, Ben Hassouna J, Rahal K. Fertility-sparing surgery in advanced stage malignant ovarian germ cell tumor: A case report. J Med Case Rep. 2017;11(1):350. doi: 10.1186/s13256-017-1516-8.
9. Gon S, Majumdar B, Barui G, Karmakar R, Bhattacharya A. Pure primary non-gestational ovarian choriocarcinoma: A diagnostic dilemma. Indian J Pathol Microbiol. 2010;53:178-80.
10. Goyal LD, Kaur S, Kawatra K. Malignant mixed germ cell tumor of ovary: An unusual combination and review of literature. J Ovarian Res. 2014;7:91. doi: 10.1186/s13048-014-0091-5.
11. Gremeau AS, Bourdel N, Kondo W, Jardon K, Canis M. Management of non-gestational ovarian choriocarcinoma: Laparoscopy can be essential. Report of two cases. Eur J Obstet Gynecol Reprod Biol. 2010;152(1):113-4. doi: 10.1016/j.ejogrb.2010.04.025.
12. Hafezi-Bakhtiari S, Morava-Protzner I, Burnell MJ, Reardon E, Colgan TJ. Choriocarcinoma arising in a serous carcinoma of ovary: An example of histopathology driving treatment. J Obstet Gynaecol Canada 2010;32(7):698-702.
13. Haruma T, Ogawa C, Nishida T, Kusumoto T, Nakamura K, Seki N, Katayama T, Hiramatsu Y. Pure choriocarcinoma of the ovary in Silver-Russell syndrome. Acta Med Okayama. 2015;69(3):183–188.
14. Hayashi S, Abe Y, Tomita S, Nakanishi Y, Miwa S, Nakajima T, Nomoto K, Tsuneyama K, Fujimori T, Imura J. Primary non-gestational pure choriocarcinoma arising in the ovary: A case report and literature review. Oncol Lett. 2015;9(5):2109-2111. doi: 10.3892/ol.2015.2985.
15. Heo EJ, Choi CH, Park JM, Lee JW, Bae DS, Kim BG. Primary ovarian choriocarcinoma mimicking ectopic pregnancy. Obstet Gynecol Sci. 2014;57(4):330-3. doi: 10.5468/ogs.2014.57.4.330.
16. Hu T, Yang M, Zhu H, Shi G, Wang H. Pure non-gestational ovarian choriocarcinoma in a 45,XO/46,XX SRY-negative true hermaphrodite. J Obstet Gynaecol Res. 2011;37:1900-1905.
17. Jain T, van Kessel K, Reed S, Paley P. Leydig cell tumor, mature teratoma, and non-gestational choriocarcinoma in a single ovary. Obstet Gynecol. 2000;95(6 Pt 2):1031. doi: 10.1016/s0029-7844(00)00876-0.
18. Jia N, Chen Y, Tao X, Ou E, Lu X, Feng W. A gestational choriocarcinoma of the ovary diagnosed by DNA polymorphic analysis: A case report and systematic review of the literature. J Ovarian Res. 2017;10(1):46. doi: 10.1186/s13048-017-0334-3.
19. Koyanagi T, Fujiwara H, Usui H, Ariga H, Machida S, Takei Y, Saga Y, Shozu M, Fukushima N, Niki T, Matsubara S, Suzuki M. Ovarian non-gestational choriocarcinoma and associated adenocarcinoma with the same germ cell origin determined by a molecular genetic approach: A case report. Pathol Int. 2016;66:529-534. doi: 10.1111/pin.12445.
20. Kumar S, Raouf ZR, Saparamadu PAM, Burney IA. Massive gastrointestinal bleeding from choriocarcinoma of the ovary. Oman Med J. 2018;33(6):527-530. doi: 10.5001/omj.2018.96.
21. Lee AC, Fong CM. Ovarian choriocarcinoma as the first manifestation of 46,XY pure gonadal dysgenesis. J Pediatr Hematol Oncol. 2011;33(1):e29-31. doi: 10.1097/MPH.0b013e3181fae731.
22. Lee AJ, Im YJ, Shim SH, Lee SJ, Kim TJ, So KA. Successful treatment of non-gestational choriocarcinoma in a 15-year-old girl: A case report. J Pediatr Adolesc Gynecol. 2021;34(2):231-233. doi: 10.1016/j.jpag.2020.11.004.
23. Lv L, Yang K, Wu H, Lou J, Peng Z. Pure choriocarcinoma of the ovary: A case report. J Gynecol Oncol. 2011;22(2):135-9. doi: 10.3802/jgo.2011.22.2.135.
24. Mascilini F, Moro F. Clinical and ultrasound features of non-gestational ovarian choriocarcinoma. Ultrasound Obstet Gynecol. 2018;52(1):121-123.
25. Nikolić B, Ljubić A, Terzić M, Arandjelović A 2^nd^, Babić S, Vucić M. Developing retroperitoneal anaplastic carcinoma with choriocarcinoma focus after ovarian non-gestational choriocarcinoma: A case report. Vojnosanit Pregl. 2012;69(12):1097-100.
26. Nishino K, Yamamoto E, Ikeda Y, Niimi K, Yamamoto T, Kajiyama H. A poor prognostic metastatic non-gestational choriocarcinoma of the ovary: A case report and the literature review. J Ovarian Res. 2021;14(1):56. doi: 10.1186/s13048-021-00810-3.
27. Ozaki Y, Shindoh N, Sumi Y, Kubota T, Katayama H. Choriocarcinoma of the ovary associated with mucinous cystadenoma. Radiat Med. 2001;19(1):55-9.
28. Park SH, Park A, Kim JY, Kwon JH, Koh SB. A case of non-gestational choriocarcinoma arising in the ovary of a postmenopausal woman. J Gynecol Oncol.;20(3):192-4. doi: 10.3802/jgo.2009.20.3.192.
29. Peng H, Li L, Bi Y. Successful management of non-gestational ovarian choriocarcinoma complicated with choriocarcinoma syndrome: A case report and a literature review. Curr Probl Cancer. 2020;44(4):100539. doi: 10.1016/j.currproblcancer.2020.100539.
30. Rao KV, Konar S, Gangadharan J, Vikas V, Sampath S. A pure non-gestational ovarian choriocarcinoma with delayed solitary brain metastases: Case report and review of the literature. J Neurosci Rural Pract. 2015;6(4):578-81. doi: 10.4103/0976-3147.169869.
31. Sadiq Q, Sekhri R, Lanjewar S. High-grade serous carcinoma of ovary with choriocarcinomatous differentiation: A case report and review of literature. J Clin Gynecol Obstet. 2020;9(3):53-59. doi: https://doi.org/10.14740/jcgo669.
32. Syed M, Meshram S, Deshpande P, Parida B. Extremely rare case of bilateral pure primary non-gestational ovarian choriocarcinoma. Pol J Radiol. 2017;82:547-550. doi: 10.12659/PJR.902578.
33. Trigueros Velázquez M, Sereno Coló JA, Villagrán Urive J. Pure form of primary ovarian choriocarcinoma: Report of a case. Ginecol Obstet Mex. 1995;63:341-5.
34. Vimala N, Kumar S, Dadhwal V. Primary choriocarcinoma of the Fallopian tube. Int J Gynaecol Obstet. 2002;79(1):37-8. doi: 10.1016/s0020-7292(02)00145-5.
35. Wan J, Li XM, Gu J. Primary choriocarcinoma of the fallopian tube: A case report and literature review. Eur J Gynaecol Oncol. 2014;35(5):604-7.
36. Wang Q, Guo C, Zou L, Wang Y, Song X, Ma Y, Liu A. Clinicopathological analysis of non-gestational ovarian choriocarcinoma: Report of two cases and review of the literature. Oncol Lett. 2016;11(4):2599-2604. doi: 10.3892/ol.2016.4257.
37. Xin L, Beier A, Tiede S, Pfiffer T, Köhler C, Favero G. Laparoscopic fertility-preserving treatment of a pure non-gestational choriocarcinoma of the ovary: Case report and review of current literature. J Minim Invasive Gynecol. 2015;22(6):1095-9.
38. Xing R, Zhou W, Zhang X, et al. A case of primary ovarian choriocarcinoma in adolescence. Chinese J Pathol. 2016,45(10):721-722. doi:10.3760/cma.j.issn.0529-5807.2016.10.014.
39. Yang Y, Zhang X, Chen D, Liu L, Hao L. Adolescent non-gestational ovarian choriocarcinoma: Report of a case and review of literature. Int J Clin Exp Pathol. 2019;12(5):1788-1794.
40. Yee LS, Zakaria R, Mohamad N, Fong OW. Non-gestational choriocarcinoma of the ovary: A case report. J Taibah Univ Med Sci. 2021;16(4):632-636. doi: 10.1016/j.jtumed.2021.01.001.
